# Using Intervention Mapping to Develop a Provider Intervention to Increase HPV Vaccination in a Federally Qualified Health Center

**DOI:** 10.3389/fpubh.2020.530596

**Published:** 2020-12-18

**Authors:** Jessica D. Austin, Serena A. Rodriguez, Lara S. Savas, Tina Megdal, Lois Ramondetta, Maria E. Fernandez

**Affiliations:** ^1^The University of Texas Health Science Center School of Public Health, Dallas Regional Campus, Dallas, TX, United States; ^2^Department of Population and Data Sciences, University of Texas Southwestern Medical Center, Dallas, TX, United States; ^3^Center for Health Promotion and Prevention Research, The University of Texas Health Science Center at Houston School of Public Health, Houston, TX, United States; ^4^Legacy Community Health, Houston, TX, United States; ^5^Department of Gynecologic Oncology and Reproductive Medicine, The University of Texas MD Anderson Cancer Center, Houston, TX, United States

**Keywords:** HPV vaccination, provider communication, intervention mapping, intervention development, cancer prevention

## Abstract

**Introduction:** A healthcare provider's ability to give a strong recommendation for the HPV vaccine is of utmost importance in increasing HPV vaccination. To reduce the burden of HPV-related cancers, there is a critical need to develop and implement theory-based interventions aimed at strengthening healthcare providers' communication about the HPV vaccine.

**Methods:** We used Intervention Mapping (IM) steps 1–5 to develop and implement a provider-level intervention that aligns with the priorities and needs of a large, urban Federally Qualified Health Center (FQHC).

**Results:** In step 1, a diverse planning group identified barriers to HPV vaccination in clinical settings and generated process maps and a logic model of the problem. Step 2 outlined outcomes and provider performance objectives of the intervention and identified knowledge, skills, self-efficacy, outcome expectations, and normative beliefs as modifiable targets that need to change for providers to deliver strong recommendations for the HPV vaccine to parents and patients. In step 3, the planning group mapped the methods of persuasive communication, information, and modeling and skills training to behavioral targets and outlined the program practical applications (strategies) components, scope, and sequence. In steps 4 and 5, the planning group produced the intervention and planned for program implementation. The iterative and participatory process of IM resulted in modifications to the initial intervention that aligned with the needs of the FQHC.

**Discussion:** IM provided a systematic, participatory, and iterative approach for developing a theory-based provider-level intervention aimed at strengthening healthcare providers' ability to provide a strong recommendation for the HPV vaccine to eligible patients and parents served by a large FQHC. IM assisted with the identification of behavioral targets and methods that move beyond HPV knowledge and reminders to create behavior change. IM can help researchers and planners describe the processes and rational behind developing interventions and may help to facilitate implementation in real-world clinical settings by tailoring intervention components to the needs of the population.

## Introduction

Human papillomavirus (HPV) is one of the most common sexually transmitted infections in the United States with an estimated 70% of individuals acquiring the infection at some point in their lifetimes (1, 2). Persistent infection with a high-risk HPV type is the leading cause of cervical cancer and associated with the development of other cancers, such as vulvar, vaginal, and anal cancers among women, and penile and anal cancers among men (3, 4). The HPV vaccine has the potential to decrease the burden of HPV-related cancers by preventing over 90% of cancers attributed to HPV infections when presented prior to exposure (5). The Centers for Disease Control (CDC) Advisory Committee for Immunization Practices recommends that healthcare providers administer the HPV vaccine series to adolescent males and females at ages 11–12 concurrent with other recommended vaccines and completing the series prior to age 13 (6–8). The CDC also recommends catch-up HPV vaccination for individuals through age 26 who are not adequately vaccinated (8). Despite national recommendations, the uptake of the HPV vaccine as an evidence-based practice remains suboptimal, resulting in underuse and missed opportunities to prevent HPV-related cancers (9). In 2017, 66% of all 13–17 year old adolescents initiated the HPV vaccine and only 51.1% received all recommended doses, well below the Healthy People 2020 benchmark of 80% (4, 10, 11). Geographic, socioeconomic, racial, and ethnic disparities in HPV vaccine initiation and completion have also emerged, further exacerbating HPV-related cancer burdens (12–17).

Studies assessing HPV vaccine uptake consistently highlight the importance of healthcare providers' recommendations (18–20). Receiving a provider recommendation is significantly associated with HPV vaccine uptake, and a provider's ability to provide a strong recommendation is considered one of the most important strategies to increase HPV vaccine coverage (11, 17–23). However, providers often fail to recommend the HPV vaccine, and they do not recommend the HPV as consistently as other vaccinations recommended for 11–12 year old adolescents (23). A number of studies have identified challenges to recommending and communicating about the HPV vaccine, such as a lack of clarity around clinical guidelines, discomfort discussing the topic with patients and parents, and a lack of confidence responding to vaccine hesitant parents (24–27). Increasing healthcare providers' knowledge, communication skills to deliver a strong recommendation, and confidence in addressing parental and patient concerns is imperative to improve uptake and coverage.

Despite being one of the strongest predictors of HPV vaccination uptake and coverage, few interventions focus on improving HPV vaccine communication. There is a need for theory-based interventions aimed at strengthening healthcare providers' communication about HPV vaccination for children. Provider-targeted interventions are often limited to delivering provider education about the vaccine or alerts in electronic medical records (20). Importantly, absent from the literature is the use of theory to inform the development and implementation of provider-level interventions. To increase HPV vaccine recommendation behaviors, intervention developers should utilize theory that supports behavior change and should make decisions about intervention components and messages using a logical and systematic approach (28–30). These interventions should also address multiple determinants, such as self-efficacy and attitudes, and align with the priority populations needs and intervention context (31–34).

IM is a theory-driven planning framework that provides a systematic process and detailed protocol for effective multi-level intervention development, implementation, and evaluation (34, 35). IM incorporates prominent health behavior theories, such of Social Cognitive Theory, to understand determinants driving a health problem and to maps methods of behavior change, such as skills training with guided practice or modeling. IM has been used to develop multiple cancer prevention and control programs to increase HPV vaccination (36, 37), and cervical, breast, and colorectal cancer screening (38–44). This paper describes the use of Intervention Mapping (IM) to systematically develop and implement a theory-based intervention to strengthen providers' recommendation for the HPV vaccine. The provider-level intervention described in this paper was one component of a larger multi-level intervention to comprehensively address factors influencing HPV vaccination in a large, urban Federally Qualified Health Center (FQHC) in Texas.

## Methods

FQHC leaders collaborated with researchers to develop a multi-level intervention to increase the proportion of FQHC age-eligible patients (11–26 years) initiating and completing the HPV vaccine series in accordance with Advisory Committee on Immunization Practice Guidelines. The overall program goal was to increase the percentage of age-eligible patients initiating the HPV vaccine within 1 year to 30%. For this paper, we describe the use of IM to develop a provider-level intervention aimed at strengthening FQHC providers' recommendation for the HPV vaccine. We define providers as physicians, nurses, medical assistants, and other medical professionals who speak about vaccination with patients and/or parents during in-person clinic visits. This study was approved by the University of Texas Health Science Center at Houston Institutional Review Board.

IM provides a systematic framework for program development and planning that can help increase the use of effective practices in healthcare settings (45–48). IM guides program planners to consider, through a needs and assets assessment, the determinants, mechanisms, and strategies for effecting change, and it encourages a particpatory approach throughout intervention development, implementation, adapatation, and dissemination (35). The IM process is composed of six steps; each one involves specific tasks that guide the translation of relevant determinants into a health promotion program. We describe the first five steps where the deliverable following the completion of each step serves as a guide for the subsequent steps (35). Step 1 includes conducting a needs assessment often led by a planning group. In step 2, planners state desired health promoting behaviors and identify performance objectives, the specific sub-behaviors required to achieve the behaviors, identify determinants associated with the health promoting behaviors, and develop a matrix of change objectives. The change objectives are the changes needed in each determinant in order for an individual to complete a performance objective and ultimately the overall health promoting behavior. To develop program components in step 3, planners identify theoretical change methods targeting determinants and operationalize methods as practical applications. Change methods are theory- or evidence-based techniques meant to influence determinants, and practical applications are ways of organizing, operationalizing, and delivering the methods (35). Program materials are produced in step 4, and step 5 includes developing an implementation plan for the intervention.

In our study for step 1, we formed a planning group and stated the current behaviors and determinants associated with failures in providing strong recommendations for HPV vaccinations to parents of HPV-vaccine eligible patients. In step 2, we stated the desired intervention outcomes, specified the provider health promoting behavior (making a strong recommendation) and performance objectives necessary to achieve the behavior, and identified psychosocial factors that influence strong provider recommendations (determinants) (34, 35). Performance objectives were crossed with determinants to create a matrix of change objectives which were a blueprint for intervention materials. In step 3, we designed the provider intervention by developing program components, mapping change objectives and determinants to theoretical change methods, and selecting practical applications, or strategies, to deliver the theoretical methods. We produced program messages and materials for providers in step 4 and, in step 5, we planned for intervention implementation by identifying who would deliver the intervention, specifying tasks necessary for intervention implementation and delivery, and developing strategies to enable implementation and delivery. Throughout each IM step, we relied on behavioral and organizational theory, evidence in the literature, and new data to the guide decision-making.

## Results

### Step 1. Logic Model of the Problem

**Figure 1:**
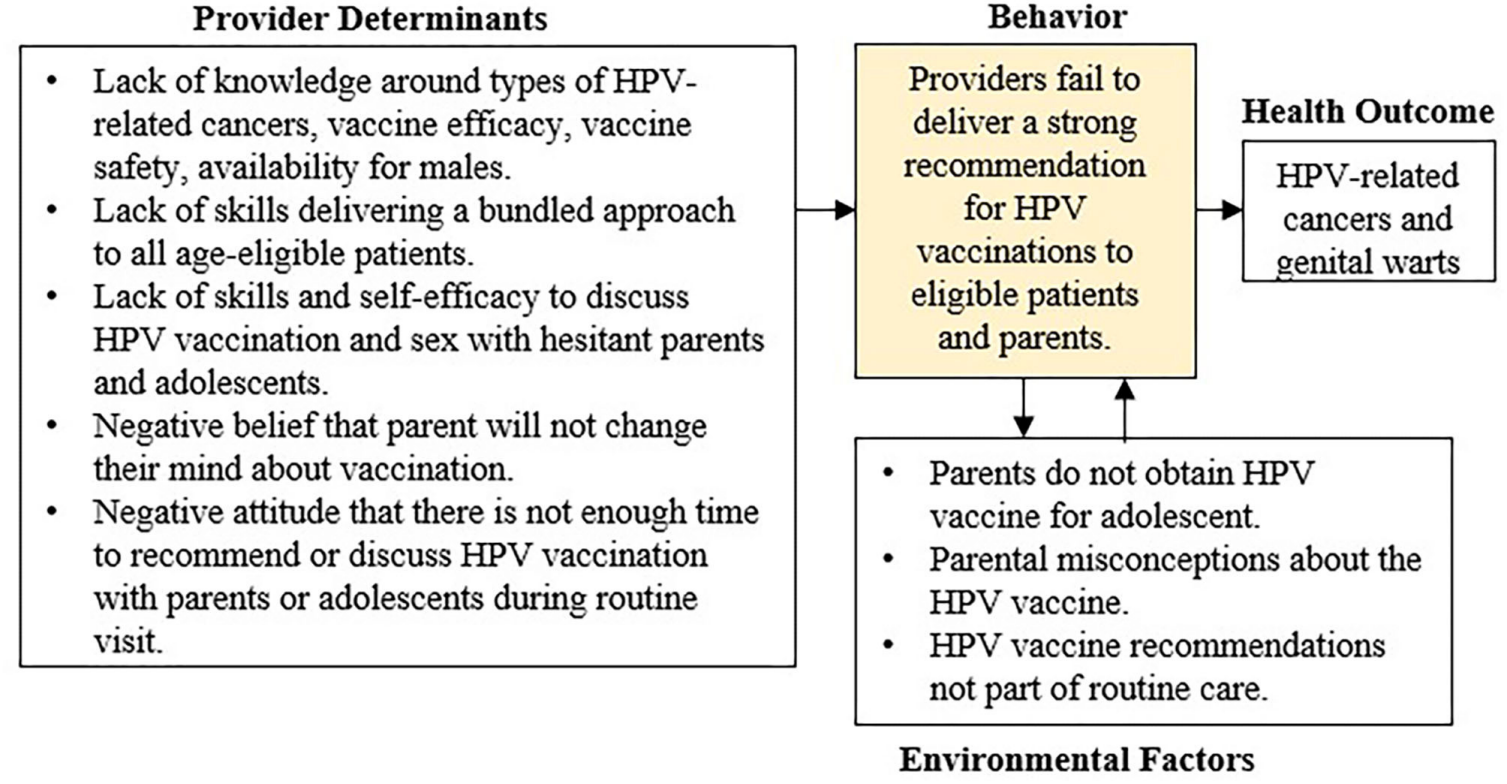
Logic model of the problem.

We established a diverse planning group comprised of stakeholders with expertise in cancer prevention and control, intervention development, HPV, and FQHC leadership. Specifically, our planning group included FQHC program leaders, 3 intervention design experts, cancer prevention and control researchers, a gynecological oncologist, and 2 program staff. The planning group, led by a cancer prevention and control researcher, met weekly during the development phase and bi-weekly during the implementation phase of the provider-level intervention. Using facilitated discussion and synthesis of provider and staff surveys, the planning group identified barriers to HPV vaccine uptake and failures in FQHC providers delivering a strong recommendations for HPV vaccinations to eligible patients and parents. The group listed barriers to uptake identified through their previous research examining HPV vaccination among adolescents (49–53), and they developed a healthcare delivery process map unique to the FQHC. Process maps are useful tools for quality improvement that illustrate key individuals and activities in clinical processes, and they assist in identifying opportunities for improvement (54, 55). The group developed the process map by listing each step of the appointment process for patients from check-in to check-out, staff rolls and responsibilities during each step, and patient handoffs (36). FQHC providers and staff provided input via email and in-person discussions throughout the process to ensure accuracy. The group combined new data from the process map with previous research to develop a logic model of the problem (Figure 1), a graphic representation of the multilevel factors associated with a lack of HPV vaccine uptake (32).

### Step 2. Program Outcomes and Objectives and Logic Model of Change

#### Outcome and Performance Objectives

The behavioral outcome was stated as “FQHC providers deliver a strong recommendation for the HPV vaccine and administering the vaccine.” We identified seven performance objectives for the outcome (Table 1).

**Table 1:** Behavioral outcome and performance objectives.

**Behavioral Outcome**: FQHC providers deliver a strong recommendation for the HPV vaccine.
**Performance Objectives**
PO1 Provider accesses patient EHR to check eligibility for HPV vaccine
PO2 Provider observes alert for HPV vaccine in EHR and notes that patient needs HPV vaccine
PO3 Provider identifies adequate vaccination schedule for the patient.
PO3a Two dose schedule
PO3b Three dose schedule
PO4 Provider delivers strong HPV vaccination recommendation to all eligible patients.
PO4a Uses recommended phrasing for HPV vaccine recommendation.
PO4b Bundles HPV vaccine with Tdap and MCV vaccination in patients ages 11-12.
PO4c Avoids separating HPV vaccine recommendation
PO5 Provider uses recommended language to explain HPV vaccine to parents or patients, if asked
PO6 Provider identifies patient or parent hesitancies, if present
PO7 Provider uses recommended language to address patient or parent hesitancies, if present

*PO, Performance Objectives*.

#### Behavioral Determinants, Change Objectives, and Matrices of Change

Based on previous research, the process map, and surveys from FQHC providers and staff, we identified the following determinants, or cognitive factors that would need to change to achieve the outcome: (1) knowledge; (2) skills and self-efficacy; (3) outcome expectations; and (4) normative beliefs. For example, behavior change models such as Social Cognitive Theory suggest that self-efficacy is a direct and proximal determinant of behavior change and is most predictive of behavioral outcomes (56–58). To increase an individual's self-efficacy, one must have the knowledge and skills to perform the behavior. Studies have shown that providers often lack knowledge around guidelines and do not have the skills to deliver strong and consistent HPV recommendations to eligible patients (20, 26). Moreover, direct feedback from FQHC staff confirmed the need for more education and training around HPV vaccination guidelines for eligible patients. Next, we crossed the determinants with performance objectives to generate a matrix of change objectives that served as the blueprint for designing the HPV provider intervention (Table 2).

**Table 2:** Partial matrix of change for behavioral outcome.

**Performance Objectives**	**Determinants**			
**FQHC providers will:**	**Knowledge**	**Skills and Self-efficacy**	**Outcome Expectations**	**Normative Beliefs**
PO4. Deliver strong HPV vaccination recommendation to all eligible patients PO4a. Uses recommended phrasing for HPV vaccine recommendation PO4b. Bundles HPV vaccine with pediatric patients Tdap and MCV vaccination PO4c. Avoids separating HPV vaccine recommendation	K4a. Describe CDC recommendation that providers deliver strong HPV vaccine recommendation K4b. Describe the components of a strong HPV vaccine recommendation K4c. Describe CDC recommendation to provide a bundled recommendation if pediatric patients patient due for other vaccines K4d. Describe that receiving a strong or bundled recommendation for the HPV vaccine is a significant predictor of vaccine uptake	SSE4a. Demonstrate ability to deliver a strong HPV vaccine recommendation SSE4b. Express confidence in ability to deliver a strong HPV vaccine recommendation SSE4c. Demonstrate ability to deliver a bundled vaccine recommendation to patients due for other vaccines SSE4d. Express confidence in ability to deliver a bundled vaccine recommendation to patients due for other vaccines	OE4a. Expect that delivering a strong HPV vaccine recommendation will reduce parental or patient hesitancy and refusal of vaccine OE4b. Expect that delivering a bundled vaccine recommendation to patients due for other vaccines will reduce parental or patient hesitancy and refusal of the HPV vaccine	NB4a. Recognize that delivering a strong HPV vaccine recommendation is recommended by the CDC NB4b. Recognize that delivering a bundled vaccine recommendation for patients due for other vaccines is recommended by the CDC NB4c. Recognize that delivering strong and bundled recommendations supports the FQHC's commitment to increasing vaccination rates among all eligible patients
PO5. Use recommended language to explain HPV vaccine to parents or patients, if asked	K5a. Describe common parental questions about HPV vaccine for pediatric patients K5b. Describe common adult questions about HPV vaccine K5c. List recommended language responding to common questions about HPV vaccine for pediatric patients K5d. List recommended language responding to common questions about HPV vaccine for adults	SSE5a. Demonstrate ability to answer common parental questions about HPV vaccine for pediatric patients using recommended language SSE5b. Express confidence in ability to answer common parental questions about HPV vaccine for pediatric patients using recommended language SSE5c. Demonstrate ability to answer common questions adult ask about HPV vaccine using recommended language SSE5d. Express confidence in ability to answer common questions adult ask about HPV vaccine using recommended language	OE5a. Expect that using recommended language to respond to common parental questions can reduce parental concerns about HPV vaccine OE5b. Expect that using recommended language to respond to common adult questions can reduce concerns about HPV vaccine	NB5a. Believe that other providers are answering common questions with recommended language NB5b. Believe that using recommended language to answer questions about the HPV vaccine provides parents and patients with consistent messaging from all FQHC staff

*PO, Performance Objectives; K, Knowledge; OE, Outcome expectations; SSE, Skills and Self-efficacy; NB, Normative Beliefs*.

### Steps 3–4. Program Design and Production

#### Change Methods and Practical Applications

Using the matrix of change objectives generated in Step 2, the team developed the program components of the HPV provider training by mapping the change objectives and determinants to change methods, and selected practical applications to deliver the methods (Table 3). The team selected change methods, or theory-based techniques, from prominent health behavior theories known to influence behavioral determinants—persuasive communication (Social Cognitive Theory), giving information (Theories of Information Processing), modeling and skills training (Social Cognitive Theory). These change methods were operationalized into practical applications (strategies) that consider the real-world clinical setting and FQHC culture including healthcare providers using convincing language to describe problems related to the vaccine, understanding the guidelines for all eligible patients, and demonstrating the ability to give a strong recommendations. The team also developed surveys to assess the impact of change methods on determinants, but due to the low response rates, it was not possible to conduct analyses for hypothesis testing. However, analysis of the completed surveys provided valuable insight into HPV vaccination practices at the FQHC.

**Table 3:** Change methods, practical applications, and program components.

**Determinants**	**Change Objectives**	**Change Methods**	**Practical Application**	**Component**
Knowledge, Outcome Expectations	K1a-c. and OE.1a	Persuasive communication	Trainer uses convincing language to describe the public health problems related to HPV and describes the HPV vaccine highlighting the role that the provider plays in increasing HPV vaccination at the FQHC	Provider Training—“HPV Education Review” module
Knowledge	K1d K3a-b	Information	Trainer informs about new vaccination guidelines regarding dosage and special populations	Provider Training—“HPV Education Review” module.
Knowledge, Skills and Self Efficacy, Outcome Expectations, Normative Beliefs	K2a. K4b-f. SSE4a-d. OE4a-b. NB4a-c	Modeling and skills training	Trainer demonstrates how to give a strong HPV recommendation and providers practice giving recommendation Trainer demonstrates how to give bundling recommendations Trainer cites data highlighting that provider recommendations play a significant role in whether patients vaccinate	Provider Training—“Communication and Skill Building” module

*K, Knowledge; OE, Outcome expectations; SSE, Skills and Self-efficacy; NB, Normative Beliefs*.

#### Intervention Production

In step 4, we designed, produced, and pre-tested the components of the intervention. Program materials included detailed flowcharts, synopses and scripts, and text and image vignettes delivered via PowerPoint. The team enlisted support from cancer prevention researchers with experience designing materials for providers to produce program materials.

A gynecologic oncologist—a member of the planning group with experience conducting HPV vaccine educational sessions—delivered the in-person provider intervention, which included three components: (1) didactic instruction and education, (2) interactive role-play opportunities using tailored messages, and (3) take-home materials. The didactic educational component highlighted up-to-date research on HPV, HPV-related cancers, vaccination rates, and guidelines, and it emphasized the importance of bundled communication. Additionally, the educational component reviewed individual provider and staff roles during adolescent clinic encounters and outlined processes in place to improve HPV vaccine uptake, such as recall reminders and pre-clinic huddles. The role-playing component was designed to strengthen the communication skills required to provide a strong HPV vaccine recommendation to patients aged 11–12, 13–18, and 18–26 years in accordance with vaccination guidelines. We incorporated communication strategies that staff could use when dealing with hesitant parents and patients, as well as responses to frequently asked questions. The one time, in-person program was designed for 1 h to fit into providers work schedule and all providers also received take-home materials including a fact-sheet about HPV vaccination and clinical guidelines.

**Figure 2:**
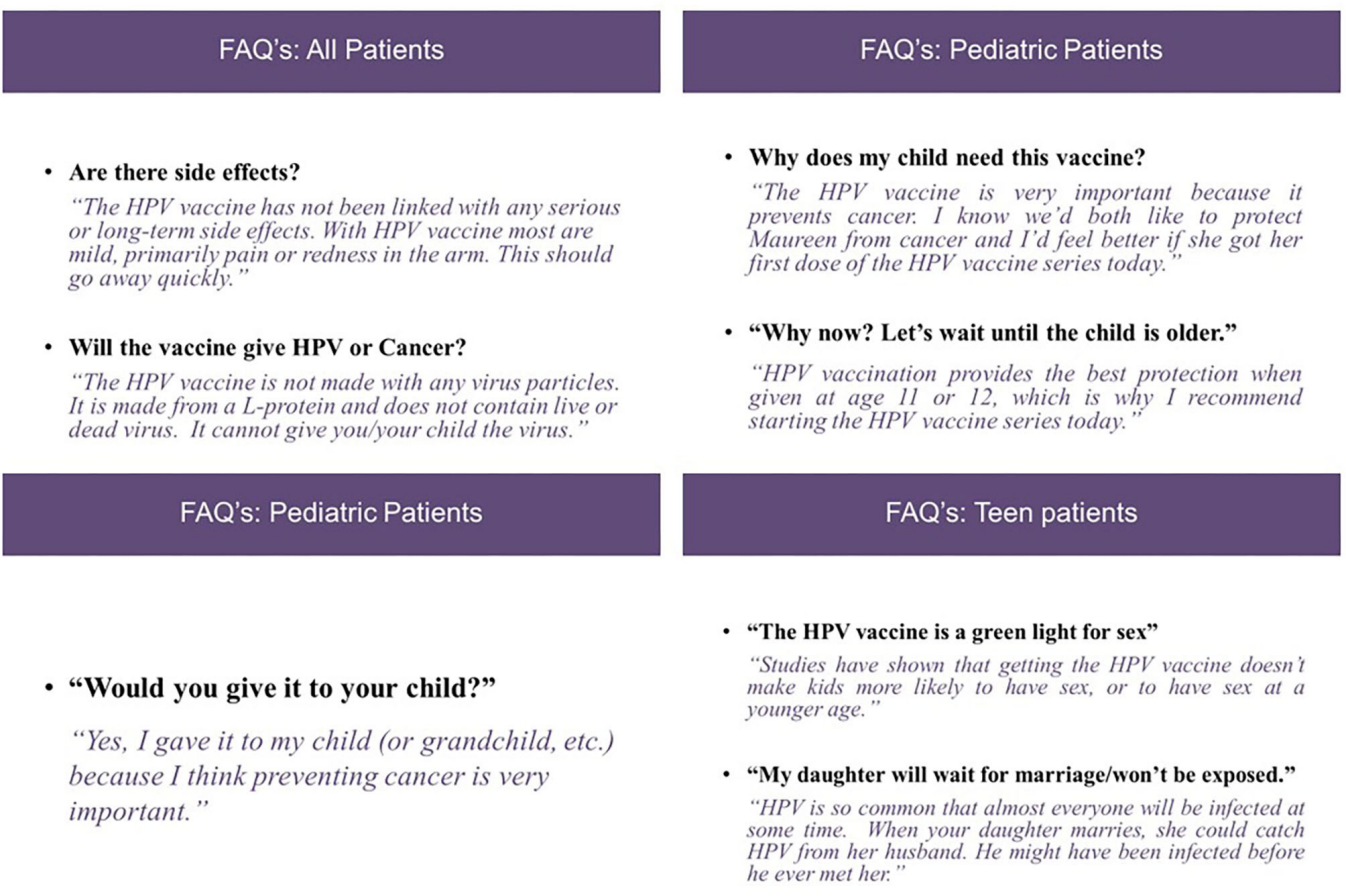
Example of provide responses to parental concerns around HPV vaccination.

Based on feedback, the in-person intervention was recorded as a webinar so that providers and staff could view the program at any time. Two versions of the webinar were audio-recorded and delivered based on provider role (physician vs. medical assistant). Figure 2 illustrates how we designed messages that included prompts for responding to eligible patients and parents who are hesitant about the vaccine.

### Step 5. Program Implementation

First, the planning group identified stakeholders for the implementation of the provider training and developed detailed summaries of the tasks that implementers would need to do to successfully implement the provider training. The planning group identified two primary implementers of the program: a clinic champion, and a gynecological oncologist/HPV vaccine advocate. The gynecological oncologist was a member of the planning group and was involved in the entire planning process. Performance objectives, or sub-behaviors that the gynecological oncologist needed to do for implementation included the following: (1) preparing for the training sessions, (2) coordinating with clinic HPV champion to schedule the in-person provider training, and (3) conducting the in-person provider training. The clinic champion implementation performance objectives included: (1) overseeing implementation efforts, (2) identifying providers who needed the training, (3) providing feedback to planning committee, and (4) interacting with the research team, FQHC providers and staff as necessary to share and address barriers to implementation and provide suggestions for overcoming these barriers. Next, we identified factors (personal determinants) influencing implementation behaviors and crossed these with performance objectives. After completing the matrix of change objectives, we identified change methods to influence the determinants of implementation behaviors and operationalized these into implementation strategies that included coordination meetings with clinic champions, gynecological oncologists training and practice presentations. Between March and August 2016, the gynecological oncologist delivered the intervention to 57 FQHC providers and staff members from four clinics including 10 physicians, 32 medical assistants, 2 nurses, and various administrative staff.

Following initial in-person sessions, we conducted interviews and surveys with staff and providers who completed the in-person training to identify areas for improvement. Providers and staff participating in the surveys and interviews requested revisions to the training in order to improve program delivery by decreasing barriers to participation be creating flexible time options for completing the training and to better tailor the content. For example, results from the interviews recommended that we redesign the presentation to fit a webinar format to minimize disruption to busy clinic schedules. Additionally, the providers requested two separate modules for providers and medical assistant and a reduction in the number of slides to decrease the overall training time in hopes of reaching a larger amount of providers. FQHC providers also requested additional information around special populations including men who have sex with men, transgender, and HIV positive individuals. Through analysis of surveys and interviews, we identified additional barriers and facilitators to HPV vaccination that we used to inform the development of case studies that demonstrate how to make a strong HPV vaccine recommendation and address common parental/adolescent concerns. These case studies were incorporated into the revised webinar training.

Participatory approaches used throughout both the planning and implementation of the intervention, also informed intervention modifications at each stage of delivery. Modifications to the intervention were based on feedback and guided by of the iterative process of IM, resulting in changes to the content (to increase relevance for the specific needs of the patient population) and delivery of the HPV provider training (to increase provider participation). For example, we updated performance objective four and incorporated new information that outlined the vaccination recommendations for all eligible age groups as well as specific populations frequently served by the FQHC including the LGBTQ community, transgender people, and people with HIV. The FQHC team agreed to distribute the webinar using their online learning management system. Between April 2017 and March 2018, 133 FQHC staff competed the webinar training.

## Discussion

Improving healthcare provider communication is one of the most highly prioritized goals in the national movement to increase HPV vaccination rates (59). Yet, healthcare providers continue to face substantial challenges delivering strong recommendations for the HPV vaccine (26). Studies consistently recommend education and support to improve provider knowledge, skills, and comfort related to discussing the HPV vaccination with patients and parents (20, 26, 28). Yet, few published studies describe the processes used to develop interventions or incorporate theory and participatory approaches throughout the development process (60–63). Theory-driven interventions that go beyond providing information to increase knowledge or embedding cues in electronic medical records are needed to support providers in recommending HPV vaccination effectively and efficiently in a highly complex communication environment (20, 64).

In this article, we provided a comprehensive and detailed description of how we used IM to systematically develop a theory-based HPV provider intervention tailored to the needs and preferences of an FQHC. Using IM, we were able to identify and establish sub-behaviors needed to achieve the behavioral outcome and provided a blueprint to map methods and strategies to address multiple determinants derived from theory, including knowledge, skills, self-efficacy, outcome expectations, and normative beliefs. IM encourages participatory approaches to engage stakeholders as part of an iterative process of program planning, development, and implementation (35). By explicitly reporting all decisions and considerations throughout the interventions process, IM makes intervention development and planning transparent. Though beyond the scope of this paper, the FQHC did surpass its overall goal of increasing the percentage of age-eligible FQHC patients who initiate the HPV vaccine within 1 year by 7%.

The iterative and participatory process of IM may facilitate the implementation to real-world setting and support reach a larger provider audience. FQHC staff and leadership, responsible for implementing the intervention, served as members of the planning committee and played a critical role throughout the process by ensuring that the intervention addressed the needs and fit of the organization. Having staff and leadership directly involved in the process played a critical role in modifying the intervention to fit the population and demonstrated the organizations commitment which, in turn, may increase sustainability (65). We acknowledge that participatory approaches can be challenging, especially when engaging clinic leaderships and providers. Providers have many responsibilities and are not always able to take time away from the office in planning meetings, especially unpaid. We addressed this challenge by working in collaboration with clinic managers and conducting interviews and surveys with FQHC clinic staff and providers who completed the training to identify areas for improvement that resulted in revisions to the format and delivery of the intervention. The systematic and iterative process of IM allowed the team to develop and revise matrices that can continue to serve as a tool for studying and understanding the potential causal mechanisms of the intervention and for comparing approaches across interventions.

There are limitations to our development process using IM. While tailoring to the FQHCs data and delivery platforms improved reach and relevance, this may limit broader intervention scale-up to other clinics and organizations. Additionally, the provider training was designed for providers serving low income, minority communities and may not be effective in other settings since materials may not resonate for other race/ethnicity populations. However, IM provides a blueprint that allows for adaptations to different populations and settings by retaining core program components (34). Further, the ability to develop and tailor intervention components to specific context may promote the uptake of the intervention by addressing the unique needs and avoiding implementation errors (66). The iterative and participatory process of IM resulted in changes to the program delivery and the removal of change methods that may be salient to behavior change, potentially reducing effectiveness. While the multi-level intervention was successful in increasing HPV vaccination at the FQHC, we did not perform an evaluation specifically on the HPV provider intervention due to low response rates on provider surveys resulting from lack of time. As a result, it is unclear to what extent the provider intervention contributed to increased HPV vaccination rates. However, analysis of completed surveys did provide data used to identify additional barriers experienced by FQHC staff when vaccinating pediatric and adult populations.

## Conclusion

Improving provider communication remains a prioritized goal to increase HPV vaccination coverage. This paper may provide useful insights for researchers, planners, and organizations interested in developing provider communication interventions in highly complex environments by providing a systematic framework that researchers can use to report the processes and rationale behind developing interventions. As a result, the process of IM may help to advance the development of HPV interventions at multiple levels by aiding in the interpretation of intervention findings, helping to identify causal mechanisms driving change, and assisting with adaptation by identifying core components of the intervention.

## Data Availability Statement

The raw data supporting the conclusions of this article will be made available by the authors, without undue reservation, to any qualified researcher.

## Ethics Statement

The studies involving human participants were reviewed and approved by University of Texas Health Science Center at Houston Institutional Review Board. The patients/participants provided their written informed consent to participate in this study.

## Author Contributions

MF, LS, and LR: study and manuscript conceptualization. JA: background, methods, results, and discussion. SR, LS, LR, and MF: contributed to background, methods, results, and discussion. TM: contributed to methods. All authors: contributed to the article and approved the submitted version.

## Conflict of Interest

The authors declare that the research was conducted in the absence of any commercial or financial relationships that could be construed as a potential conflict of interest.
